# Conversion Surgery for Advanced Gastric Cancer with Para-Aortic Lymph Node Metastases Following Treatment with Capecitabine, Oxaliplatin, and Pembrolizumab: A Case Report

**DOI:** 10.70352/scrj.cr.25-0622

**Published:** 2026-01-14

**Authors:** Takuya Ono, Yuhei Waki, Masumi Takamoto, Kazunori Tokuda, Koichi Sato, Atsushi Horiuchi

**Affiliations:** Department of Gastrointestinal Surgery, Ehime Prefectural Niihama Hospital, Niihama, Ehime, Japan

**Keywords:** gastric cancer, pembrolizumab, immune checkpoint inhibitor, conversion surgery

## Abstract

**INTRODUCTION:**

Immune checkpoint inhibitors (ICIs) have recently emerged as an important treatment option for various cancers. In 2024, pembrolizumab was approved as a first-line treatment for unresectable or recurrent gastric cancer. Conversion surgery following ICI-based chemotherapy has been reported; however, cases involving pembrolizumab-based first-line therapy remain rare. Here, we report a case of conversion surgery after treatment with capecitabine plus oxaliplatin (CAPOX), combined with pembrolizumab for unresectable advanced gastric cancer.

**CASE PRESENTATION:**

An 82-year-old man presented with anorexia and was referred to our department for surgical evaluation. Upper gastrointestinal endoscopy revealed circumferential type 3 gastric cancer extending from the lower gastric body to the antrum with pyloric stenosis. Contrast-enhanced CT showed para-aortic lymph node metastases, resulting in a diagnosis of stage IVB gastric cancer (cT4aN2M1, 15th edition of the Japanese Classification of Gastric Carcinoma [JGCA]). First, we performed a laparoscopic gastrojejunal bypass to treat anorexia and oral intake difficulties due to pyloric stenosis. The patient then received 5 cycles of CAPOX plus pembrolizumab. Subsequent upper gastrointestinal endoscopy revealed significant scarring with residual cancer cells, and contrast-enhanced CT showed significant shrinkage of the primary tumor lesion and para-aortic lymph nodes. Because R0 resection was achievable, we performed conversion surgery involving open distal gastrectomy with D2 and para-aortic lymphadenectomy. Postoperative pathological findings revealed a small number of residual cancer cells in the submucosa, with no viable cancer cells detected in the para-aortic lymph nodes (ypT1bN0M0, ypStage IA). The pathological response grade was 2b according to the 15th edition of the JGCA. At 6 months postoperatively, the patient remains alive and recurrence-free.

**CONCLUSIONS:**

Conversion surgery after CAPOX plus pembrolizumab chemotherapy is a potential therapeutic strategy for unresectable advanced gastric cancer.

## Abbreviations

CA19-9
: carbohydrate antigen 19-9
CAPOX
: capecitabine plus oxaliplatin
CEA
: carcinoembryonic antigen
CPS
: combined positive score
CR
: complete response
HER2
: human epidermal growth factor receptor 2
ICI
: immune checkpoint inhibitors
irAEs
: immune-related adverse events
JGCA
: Japanese Classification of Gastric Carcinoma
MSI-high
: microsatellite instability-high
MST
: median survival time
ORR
: overall response rate
OS
: overall survival
PFS
: progression-free survival
PR
: partial response

## INTRODUCTION

Recently, advances in chemotherapy, molecular targeted therapies, and immune checkpoint inhibitors (ICIs) have improved the prognosis of patients with advanced gastric cancer. Trastuzumab, a human epidermal growth factor receptor 2 (HER2)–targeted agent, was the first molecular therapy developed for unresectable advanced or recurrent gastric cancer and has become the standard first-line treatment, significantly improving the overall survival (OS) of patients with unresectable or recurrent HER2-positive gastric cancer.^1)^

For patients with HER2-negative, unresectable, or recurrent gastric cancer, recent clinical trials involving combinations of chemotherapy with ICIs have shown promising therapeutic efficacy, and these combinations have become standard treatments in Japan.^2–4)^ Based on the survival benefits shown in the ATTRACTION-4 and CheckMate 649 trials, chemotherapy combined with nivolumab was approved in November 2021 as a first-line treatment for HER2-negative unresectable advanced or recurrent gastric cancer.^2,3)^ Pembrolizumab monotherapy was recommended in the Japanese Gastric Cancer Treatment Guidelines 2021 as second-line or later treatment for gastric cancer with microsatellite instability (MSI)-high based on the results of the KEYNOTE-158 and KEYNOTE-061 trials.^5–7)^ In the KEYNOTE-859 trial, the superiority of pembrolizumab plus chemotherapy over chemotherapy alone in the first-line treatment of HER2-negative unresectable or recurrent gastric cancer was examined, and the results showed that pembrolizumab plus chemotherapy significantly extended OS and progression-free survival (PFS) compared with that using chemotherapy alone.^4)^ In Japan, based on these trial results, pembrolizumab plus chemotherapy was approved as a first-line treatment for unresectable advanced or recurrent gastric cancer in May 2024. However, despite recent advances in chemotherapy, gastric cancer prognosis remains unfavorable, and further improvements are needed in treatment strategies for unresectable or recurrent gastric cancer.

Conversion surgery, designed for stage IV gastric cancer, may improve long-term survival. It is defined as a surgical treatment aimed at achieving R0 resection following systemic chemotherapy for tumors that were originally unresectable for technical and/or oncological reasons.^8,9)^ The CONVO-GC-1 classification system is used to guide treatment strategies in stage IV gastric cancer and provides an important criterion when attempting conversion surgery.^10,11)^ Category 1 included technically resectable metastases; category 2, marginally resectable metastases; category 3, peritoneal dissemination; and category 4, peritoneal dissemination and metastasis to other organs. Conversion surgery is typically considered for patients in categories 1, 2, or 3 who achieve a satisfactory response to chemotherapy.^10)^

Conversion surgery after ICI-based chemotherapy has been reported; however, cases involving pembrolizumab-based therapy for unresectable advanced gastric cancer remain limited. Herein, we report a case in which conversion surgery was performed after effective capecitabine plus oxaliplatin (CAPOX) combined with pembrolizumab therapy for unresectable advanced gastric cancer.

## CASE PRESENTATION

**Fig. 1:**
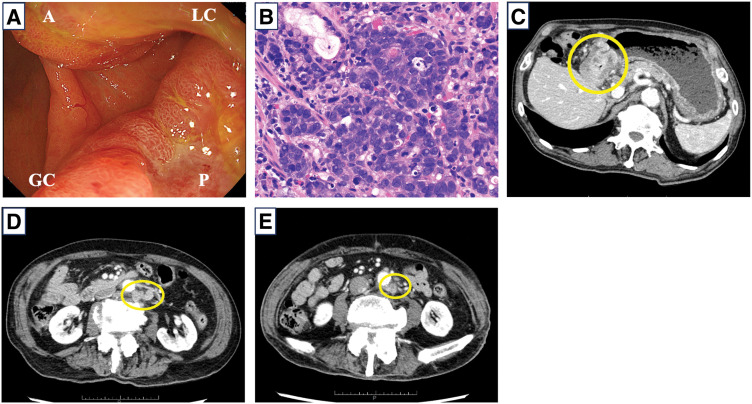
Upper gastrointestinal endoscopic, pathological findings, and contrast-enhanced CT before chemotherapy. (**A**) Upper gastrointestinal endoscopy showing circumferential stenosis in the lower gastric body due to a type 3 tumor. (**B**) A biopsy revealed differentiated adenocarcinoma (por1). (**C**) Contrast-enhanced CT showing circumferential wall thickening at the tumor site. (**D**, **E**) Para-aortic lymph nodes nos. 16b1 (**D**) and 16b2 (**E**) were enlarged (circles). A, anterior wall; GC, greater curvature; LC, lesser curvature; P, posterior wall

An 82-year-old man initially presented to his previous physician with anorexia. Upper gastrointestinal endoscopy revealed circumferential stenosis in the lower gastric body due to a type 3 tumor with duodenal bulb infiltration (**Fig. 1A**). He was referred to our department for further examination and treatment. Biopsy confirmed poorly differentiated adenocarcinoma (por1) (**Fig. 1B**). Blood tests showed carcinoembryonic antigen (CEA) and carbohydrate antigen 19-9 (CA19-9) levels within normal range (CEA 5.2 ng/mL, CA19-9 below 2.06 U/mL), with no significant increase in tumor markers.

Contrast-enhanced CT revealed circumferential wall thickening of the lower gastric body, with wall deformation and serosal surface irregularities, suggesting invasion to the serosa (**Fig. 1C**). Swelling of the infrapyloric and para-aortic lymph nodes (nos. 16a2 lat, b1 int/lat, and b2 lat) was also observed (**Fig. 1D**). The patient was diagnosed with unresectable advanced gastric cancer: lower/distal stomach, circumferential, Bormann type 3, cT4aN2M1 (with lymph node metastases [LYM]), cStage IVB (15th edition of the Japanese Classification of Gastric Carcinoma [JGCA]). Biomarker statuses are HER2-negative, CLDN18-negative, MSI-negative, and programmed death-ligand 1 (PD-L1)-positive (combined positive score [CPS] ≥10).

**Fig. 2:**
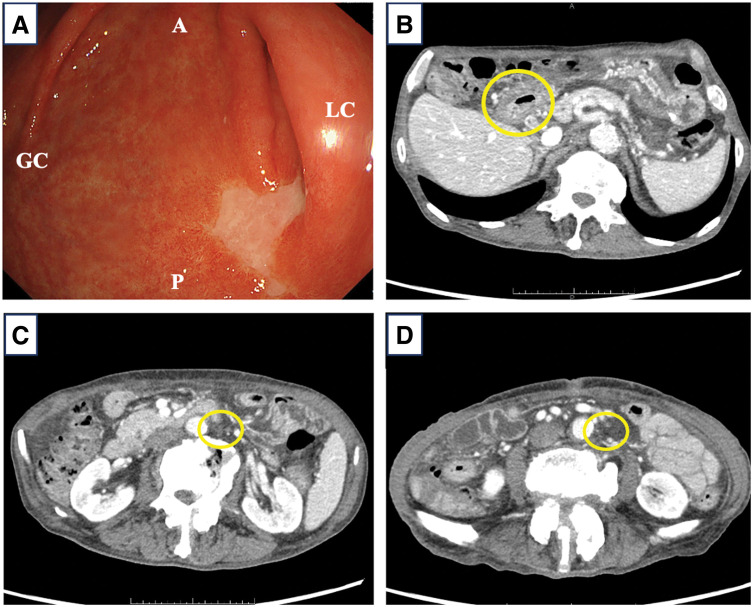
Upper gastrointestinal endoscopic and contrast-enhanced CT findings after chemotherapy. (**A**) Upper gastrointestinal endoscopy showing the tumor shrinkage and white scar. (**B**) Contrast-enhanced CT showing the main tumor shrinking. (**C**, **D**) The size of the para-aortic lymph nodes nos. 16b1 (**C**) and 16b2 (**D**) significantly decreased (circles). A, anterior wall; GC, greater curvature; LC, lesser curvature; P, posterior wall

A laparoscopic gastrojejunal bypass was performed to relieve anorexia caused by pyloric stenosis, and CAPOX plus pembrolizumab therapy was initiated. The regimen comprised pembrolizumab 200 mg/body and oxaliplatin 130 mg/m^2^ on day 1, and capecitabine 2000 mg/m^2^/day on days 1–14. Considering the patient’s advanced age, oxaliplatin and capecitabine doses were reduced by 10%–30% per cycle. No chemotherapy-related or immune-related adverse events were observed. After five cycles, upper gastrointestinal endoscopy revealed that the tumor site was mostly replaced by scar tissue (**Fig. 2A**); however, residual cancer cells were detected in the biopsy specimens. Contrast-enhanced CT showed shrinkage of the primary tumor (**Fig. 2B**), significant reduction in infrapyloric and para-aortic lymph node enlargement (**Fig. 2C**), and no new metastatic lesions. The post-chemotherapy clinical stage was ycT2N0M0, yc Stage I (partial response [PR], Response Evaluation Criteria in Solid Tumors classification). Tumor markers post-chemotherapy remained stable (CEA 7.7 ng/mL, CA19-9 <2.06 U/mL).

**Fig. 3:**
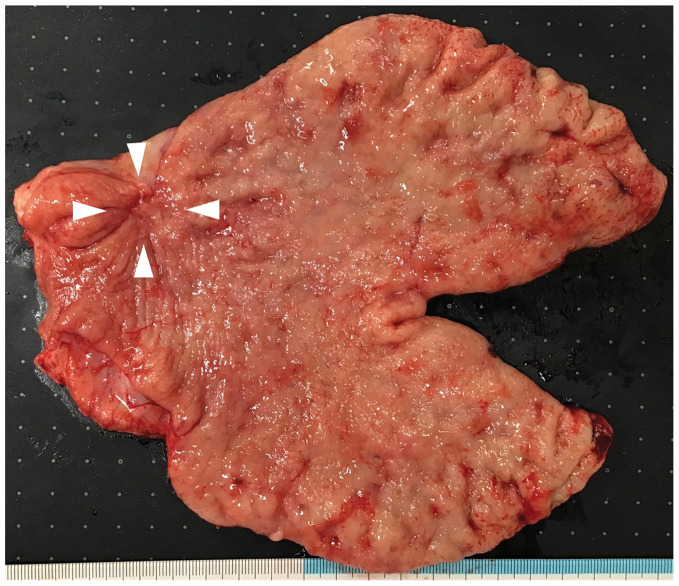
Macroscopic findings of the resected stomach. The tumor was shrinking, with a white scar observed in the lower gastric body (arrowheads).

Given these positive treatment outcomes and the absence of new metastases, we considered R0 resection feasible and performed conversion surgery. The patient underwent an open distal gastrectomy with D2 and para-aortic lymphadenectomy (16a2, b1). The operative time was 431 min, with blood loss of 682 mL. Oral food intake was resumed on POD 3, and the patient was discharged on day 11 without complications. **Figure 3** shows the resected stomach specimen, which macroscopically revealed tumor shrinkage with scarring in the lower gastric body.

**Fig. 4:**
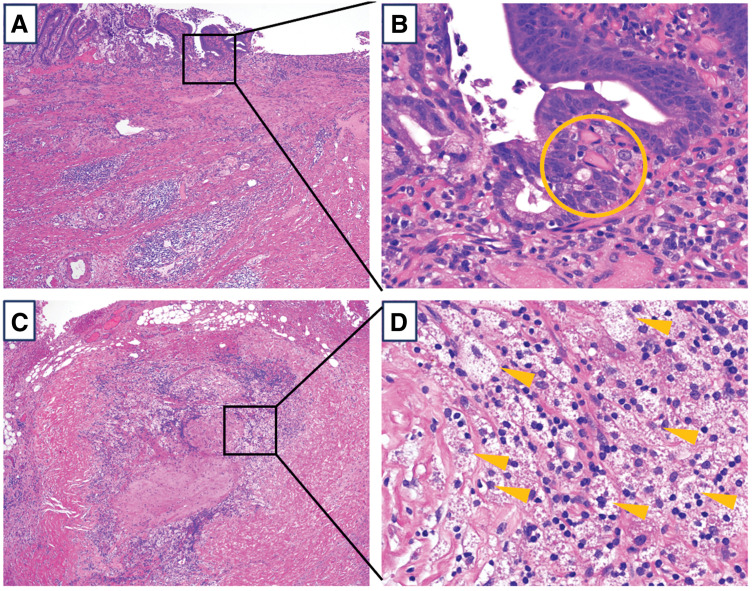
Pathological findings after conversion surgery. (**A**, **B**) Primary gastric cancer lesion. (**A**) is with a low-power field, and (**B**) is with a high-power field. The main tumor had almost disappeared, with a small number of tumor cells remaining only in the submucosa (circle). In the layers beyond the muscularis propria, the tumor cells had been replaced by macrophages and fibrotic tissue, with no viable cells remaining. (**C**, **D**) Para-aortic lymph nodes. (**C**) is with a low power field, and (**D**) is with a high power field. In the lymph nodes, tumor cells had been replaced by macrophages, with no viable tumor cells remaining (arrowheads).

Postoperative pathological findings revealed near-complete disappearance of the primary tumor, with only a few tumor cells remaining in the submucosa (**Fig. 4A**, **4B**). Macrophage clusters were observed in the infrapyloric and para-aortic lymph nodes; however, no viable cells remained (**Fig. 4C**, **4D**). The pathological diagnosis was ypT1bN0M0, yp Stage IA (15th edition of JGCA), with a pathological response grade of 2b, according to the Japanese classification (15th edition of JGCA). The patient was followed up without postoperative adjuvant chemotherapy. At 6 months post-surgery, he remains recurrence-free.

## DISCUSSION

Chemotherapy has traditionally been the standard treatment for stage IV gastric cancer in patients without acute complications from the primary tumor, such as bleeding or perforation. Recent advances in chemotherapy have made conversion surgery a feasible option for patients whose tumors were initially deemed unresectable. Several case reports suggest that conversion surgery may improve long-term survival; however, no randomized controlled trials have been conducted to compare continued chemotherapy with conversion surgery in patients with stage IV gastric cancer who respond to systemic therapy. Therefore, the role of conversion surgery in these patients remains controversial.

Recent studies have highlighted the prognostic value of conversion surgery.^11–13)^ In the CONVO-GC-1 study, outcomes were evaluated for 1206 patients with stage IV gastric cancer who underwent conversion surgery. In this study, the median survival time (MST) of the patients who achieved R0 resection was 56.6 months. A positive outcome was observed in patients with para-aortic LYM; the MST for R0 resection was 44.7 and 116.7 months in patients with nos. 16a2/b1 and 16a1/b2 metastases, respectively.^11)^ By contrast, previous clinical trials have reported MSTs of 17–18 months for patients with stage IV gastric cancer treated with chemotherapy alone.^14,15)^ These findings suggest that conversion surgery may offer a survival advantage compared with that of previous treatment strategies. In Japan, conversion surgery for stage IV gastric cancer is considered when a favorable response to chemotherapy has been achieved, and R0 resection is deemed feasible.

Several cases of conversion surgery after chemotherapy combined with nivolumab for stage IV gastric cancer have been reported; however, reports of such surgeries following first-line pembrolizumab-based therapy remain limited. In Japan, chemotherapy combined with pembrolizumab was approved in May 2024 as a first-line treatment for unresectable advanced or recurrent gastric cancer, based on the results of the KEYNOTE-859 trial. In this global, double-blind, randomized controlled phase III trial, pembrolizumab plus chemotherapy was compared with chemotherapy alone as first-line treatment of HER2-negative unresectable advanced or recurrent gastric cancer. Pembrolizumab plus chemotherapy extended OS and PFS compared with those using chemotherapy alone (median OS: 12.9 vs. 11.5 months; median PFS: 6.9 vs. 5.6 months). The overall response rate (ORR) and complete response (CR) were also higher in the pembrolizumab plus chemotherapy group than those in the chemotherapy-alone group (ORR: 51.3% vs. 42.0%; CR: 9.5% vs. 6.2%). Furthermore, the additional effect of pembrolizumab was substantial in patients with CPS ≥10 (ORR: 60.7%, CR: 12.9%).^4)^ Consequently, pembrolizumab-based chemotherapy for stage IV gastric cancer can downsize the tumor, increasing the likelihood of achieving R0 resection, suggesting a potential role for conversion surgery in improving prognosis.

Recent evidence indicates that R0 resection rates and pathological responses achieved with conversion surgery differ markedly between conventional chemotherapy and ICI-combined regimens. For example, conventional regimens such as S-1 plus cisplatin yielded R0 resection and pathological CR rates of 79.7% and 4.1%, respectively,^16)^ whereas ICI-combined chemotherapy showed higher rates, achieving 83.3% and 10.4%, respectively.^17)^ These findings suggest that incorporating an ICI may enhance preoperative tumor regression compared with using conventional chemotherapy.

PD-L1 expression in the tumor was evaluated using the CPS. At our institution, the PD-L1 Immunohistochemistry 22C3 assay is routinely used, with CPS categorized as <1, 1≤ CPS <10, and ≥10. Because the present case exhibited a CPS ≥10, we selected pembrolizumab-combined chemotherapy as first-line treatment, consistent with clinical trial data showing benefit in this population.^4)^ Both PD-1 inhibitors, nivolumab and pembrolizumab, have shown survival benefits in clinical trials; however, subtle differences exist in trial designs and reported outcomes. Subgroup analyses focused specifically on patients with CPS ≥10 were conducted only in pembrolizumab studies. While direct comparison between trials is limited by differences in trial populations and designs, in the CPS ≥10 subgroup, the hazard ratios for OS and PFS of pembrolizumab-based chemotherapy versus chemotherapy-alone (OS: 0.65; PFS: 0.62)^4)^ appear potentially more favorable than those reported for nivolumab-based chemotherapy (OS: 0.80; PFS: 0.77).^3)^

In the present case, no chemotherapy combined with ICI-related adverse events was observed during the perioperative period. However, such regimens carry not only the risks associated with conventional chemotherapy but also the potential for immune-related adverse events (irAEs). In studies of pembrolizumab-based chemotherapy, irAEs have been reported in approximately 30.8% of patients^4)^ and can occur both during chemotherapy and in the perioperative period.^18)^ These observations underscore the importance of surgeons to exercise particular caution regarding unfamiliar irAEs when performing conversion surgery.

The optimal timing of conversion surgery for stage IV gastric cancer remains undefined. Clinical studies of S-1, oxaliplatin, and nivolumab for unresectable or recurrent gastric cancer indicate that tumor reduction typically occurs within the first 4 months of treatment, with further shrinkage beyond 6 months rarely observed.^19)^ In general, based on previous studies, conversion surgery is considered after 4–6 cycles of chemotherapy. In this case, the patient achieved a PR after five cycles of CAPOX plus pembrolizumab and subsequently underwent successful R0 resection.

This patient presented with para-aortic LYM (nos. 16a2 lat, b1 int/lat, and b2 lat) and was classified as Category 2 according to the CONVO-GC-1 categorization.^11)^ A previous study reported that treatment with D2 lymphadenectomy plus para-aortic lymphadenectomy did not improve the survival rate compared with that using D2 lymphadenectomy alone in curable gastric cancer.^20)^ However, the therapeutic effect and appropriate para-aortic lymphadenectomy dissection range in patients with advanced cancer and para-aortic LYM that shrink owing to chemotherapy remain unclear and controversial. In patients with advanced gastric cancer with para-aortic LYM, several studies revealed that R0 resection following chemotherapy showed favorable long-term survivals.^11,12,21)^ In this case, contrast-enhanced CT after chemotherapy revealed resolution of the enlarged para-aortic lymph nodes. Previous studies have established the safety of para-aortic lymph node dissection for nos. 16a2 and 16b1, and R0 resection was considered achievable through systematic dissection of these nodes in this patient.^20)^ Considering both the oncologic rationale and the magnitude of surgical invasiveness, systematic para-aortic lymph node dissection of these nodes was performed to evaluate histological response to chemotherapy and assess residual tumor cells.

Currently, no standardized treatment strategy has been established regarding postoperative chemotherapy following conversion surgery. In this case, postoperative chemotherapy was not administered. This decision was based on the patient’s advanced age, the marked pathological response with a final pathological diagnosis of ypStage IA (15th edition of JGCA), and the achievement of an R0 resection, all of which suggested a potentially favorable postoperative course without additional systemic treatment.^11)^

Recently, we encountered a case in which CAPOX plus pembrolizumab therapy as first-line treatment was highly effective for unresectable advanced gastric cancer, and conversion surgery was performed. A search of PubMed using the keywords “gastric cancer,” “conversion surgery,” and “pembrolizumab” for articles published from 1968 to 2024 yielded 2 reports. In these reports, conversion surgery was performed after pembrolizumab therapy as third-line treatment. To our knowledge, this is the first case report in which conversion surgery was performed after chemotherapy with pembrolizumab as the first-line treatment for unresectable advanced gastric cancer.

The limitations of this case study include the short postoperative follow-up of 6 months, making longer-term outcomes essential to validate the durability of response after ICI-based conversion surgery. Additional evidence from prospective clinical trials is required to assess the results of conversion surgery for gastric cancer. Finally, we referred to 2 ongoing randomized controlled phase III trials, AIO-FLOT5/RENAISSANCE and JCOG2301, currently being conducted to evaluate the additional benefit of conversion surgery. In these trials, the superiority of conversion surgery after chemotherapy over chemotherapy alone will be evaluated in patients with initially unresectable gastric cancer who achieve a favorable response to first-line chemotherapy and become candidates for R0 resection.^22,23)^ The results of these trials will be important for clarifying the role of conversion surgery in patients with initially unresectable gastric cancer, and close attention should be paid to the outcomes.

## CONCLUSIONS

This case report highlights the significance of chemotherapy combined with pembrolizumab as first-line treatment for patients with unresectable advanced gastric cancer. Therefore, developing a treatment strategy that incorporates conversion surgery is essential.

## DECLARATIONS

### Availability of data and materials

All data generated or analyzed during this study are included in this published article.
